# Supplementary material for: Induced Pluripotent Stem Cell-Derived Fibroblasts Efficiently Engage Senescence Pathways but Show Increased Sensitivity to Stress Inducers
Source: Cells. 2024 May 16;13(10):849. doi: 10.3390/cells13100849 (PMC11119907; doi:10.3390/cells13100849)
Supplement: Supplementary file 1 [file cells-13-00849-s001.zip › cells-3005198-supplementary.pdf]

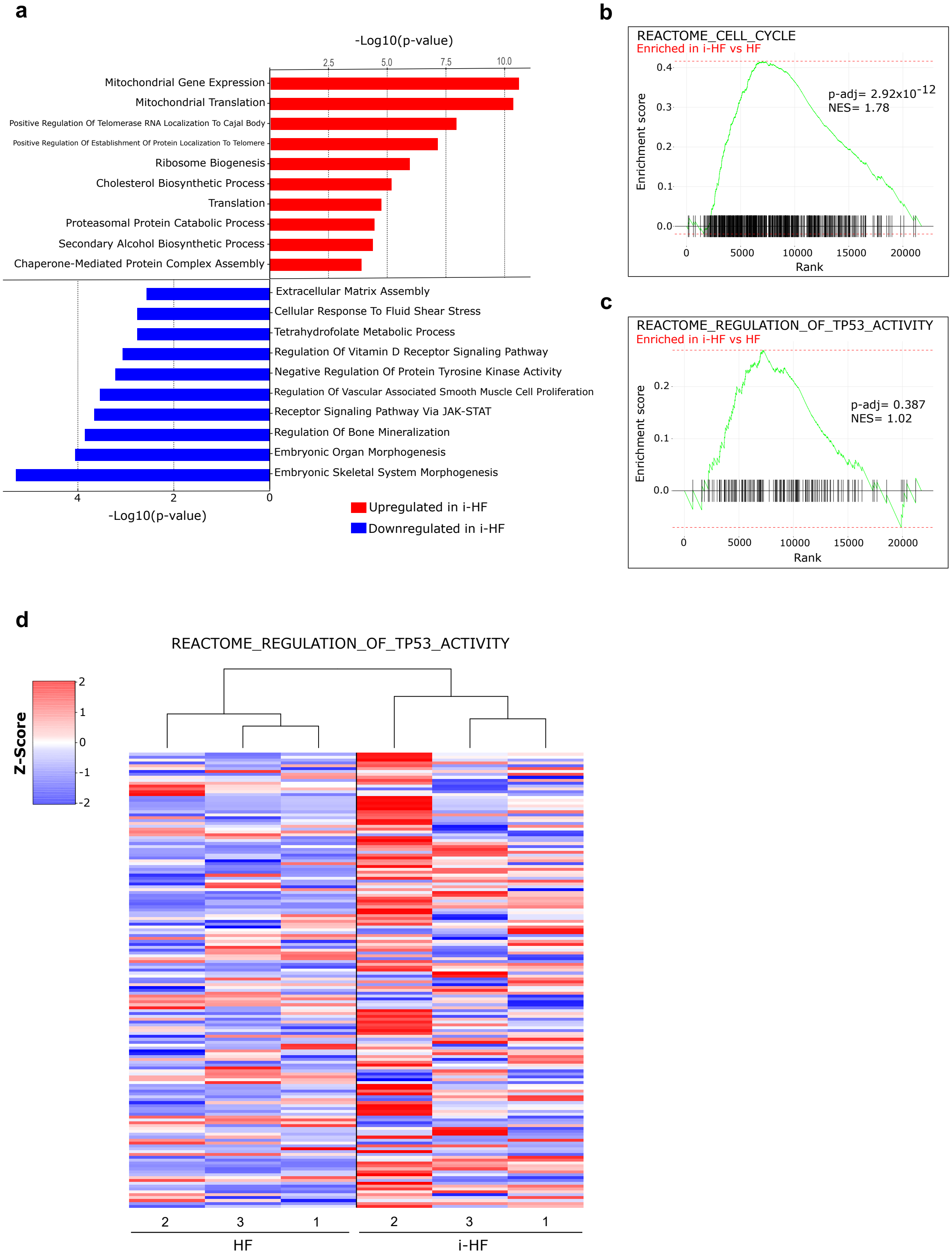

Figure S1. Transcriptional analysis of HF and i-HF. A) Gene Ontology (GO) enrichment analysis based on up- or downregulation of biological processes terms from differentially expressed genes (DEG) found in i-HF compared to HF of the three different donors. B-C) Gene set enrichment analysis (GSEA) were conducted on the pool of the three donors associated with the indicated biological pathways in i-HF compared to HF are shown. P-adj=Adjusted p-value, NES=Normalized enrichment score. D) Heatmap illustrating the transcriptional landscape associated with the regulation of TP53 activity gene set for each cell line. Color scale reflects the standard deviation of gene expression level with upregulation in red and downregulation in blue. Hierarchical clustering indicates level of similarities among samples.

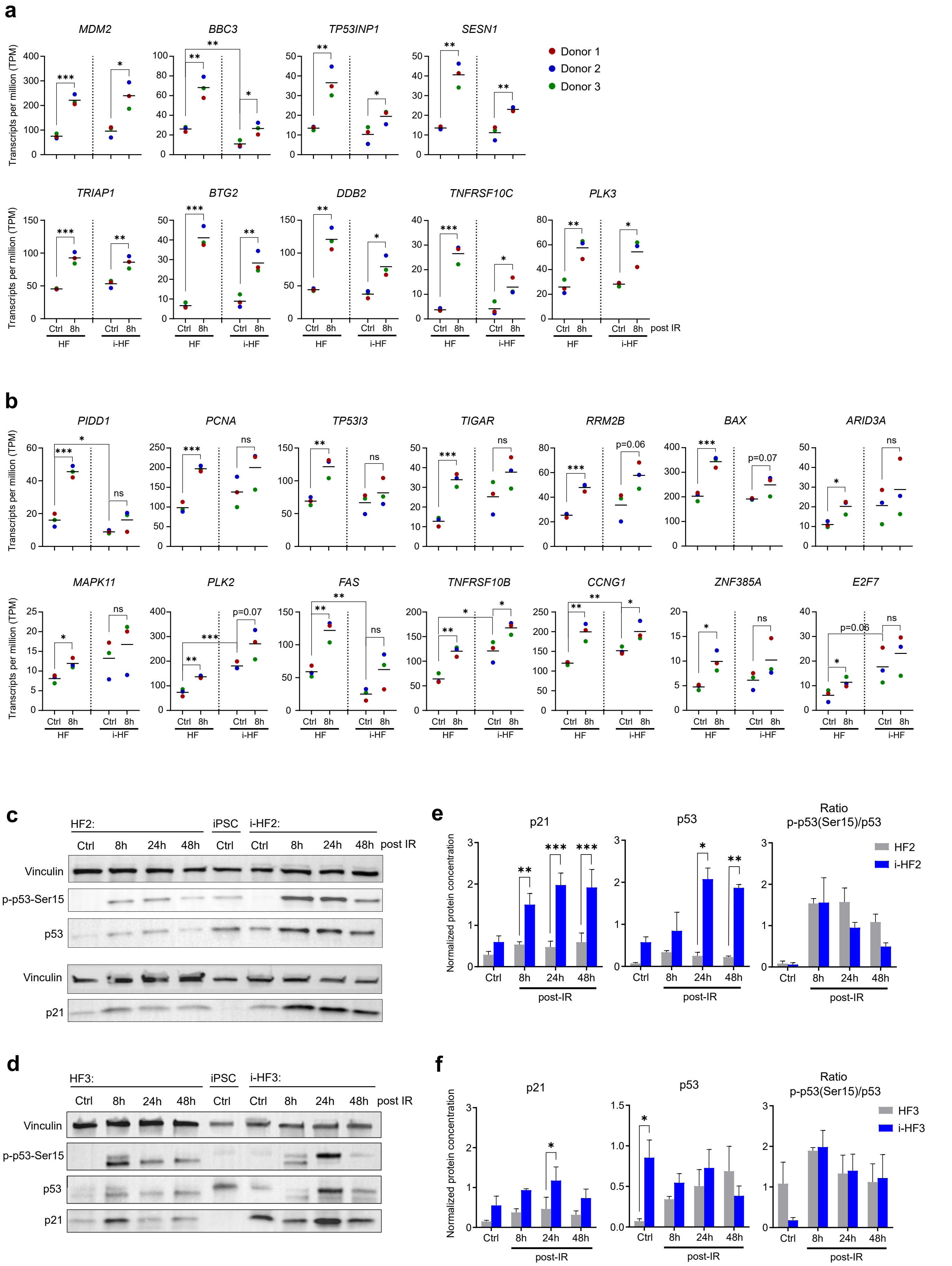
a
MDM2
BBC3
TP53INP1
SESN1
Donor 1
Donor 2
Donor 3
TRIAP1
BTG2
DDB2
TNFRSF10C
PLK3
Transcripts per million (TPM)
Ctrl
8h
post IR
HF
i-HF
b
PIDD1
PCNA
TP53I3
TIGAR
RRM2B
BAX
ARID3A
MAPK11
PLK2
FAS
TNFRSF10B
CCNG1
ZNF385A
E2F7
p=0.06
p=0.07
ns
c
HF2:
iPSC
i-HF2:
Ctrl
8h
24h
48h
post IR
Vinculin
p-p53-Ser15
p53
p21
d
HF3:
iPSC
i-HF3:
e
p21
p53
Ratio
p-p53(Ser15)/p53
HF2
i-HF2
Normalized protein concentration
post-IR
f
HF3
i-HF3

Figure S2. p53-related effectors gene and protein expression in HF and i-HF following IR. A-B) Number of mRNA transcripts per million (TPM) mapped reads from RNA-seq data of each of the three donors of HF and i-HF at basal level (Ctrl) and 8h post exposure to 15 Gy IR. Shown are the DEG common for both cell line or only in HF as shown in the Venn diagram from figure 2B. Lines represent the mean value. Data was analyzed using multiple unpaired t tests between HF and i-HF for each group. C-D) Representative western blot membranes showing protein expression of p21, p53 and phosphorylated p53 at serine 15 (p-p53(Ser15)) from clone 1 of HF and i-HF donor 2 and 3 after 8, 24 and 48h exposure to 15 Gy IR. Vinculin was used as a loading control. E-F) Quantification of p21 and p53 protein expression normalized on vinculin signal with the quantified ratio of p-p53(Ser15) on total p53 protein signal of donor 2 and 3 respectively. Data analysis was performed using a two-way ANOVA followed by a Fisher's LSD post-hoc test. Shown is mean±SEM of n=3 individual western blots.

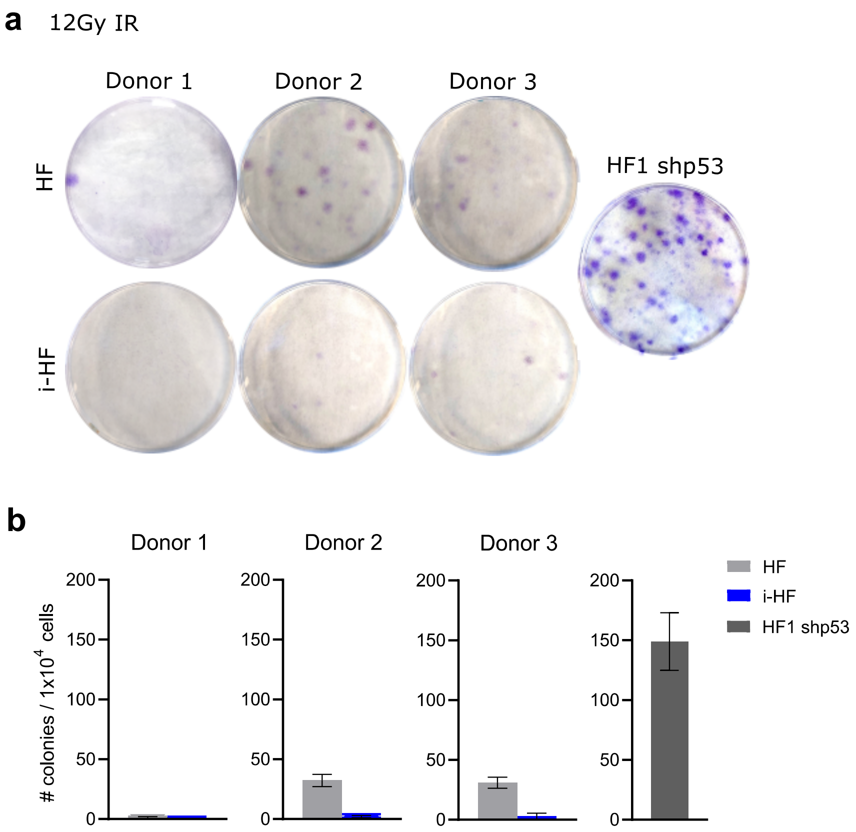

Figure S3. I-HF cell lines are more sensitive to IR. A) Representative images showing the results of a colony formation assay on cells exposed or not to 12 Gy IR. B) Quantification of the number of colonies counted following senescence induction on cell lines described in panel A. Shown is mean±SD from 3 replicates of one experiment.

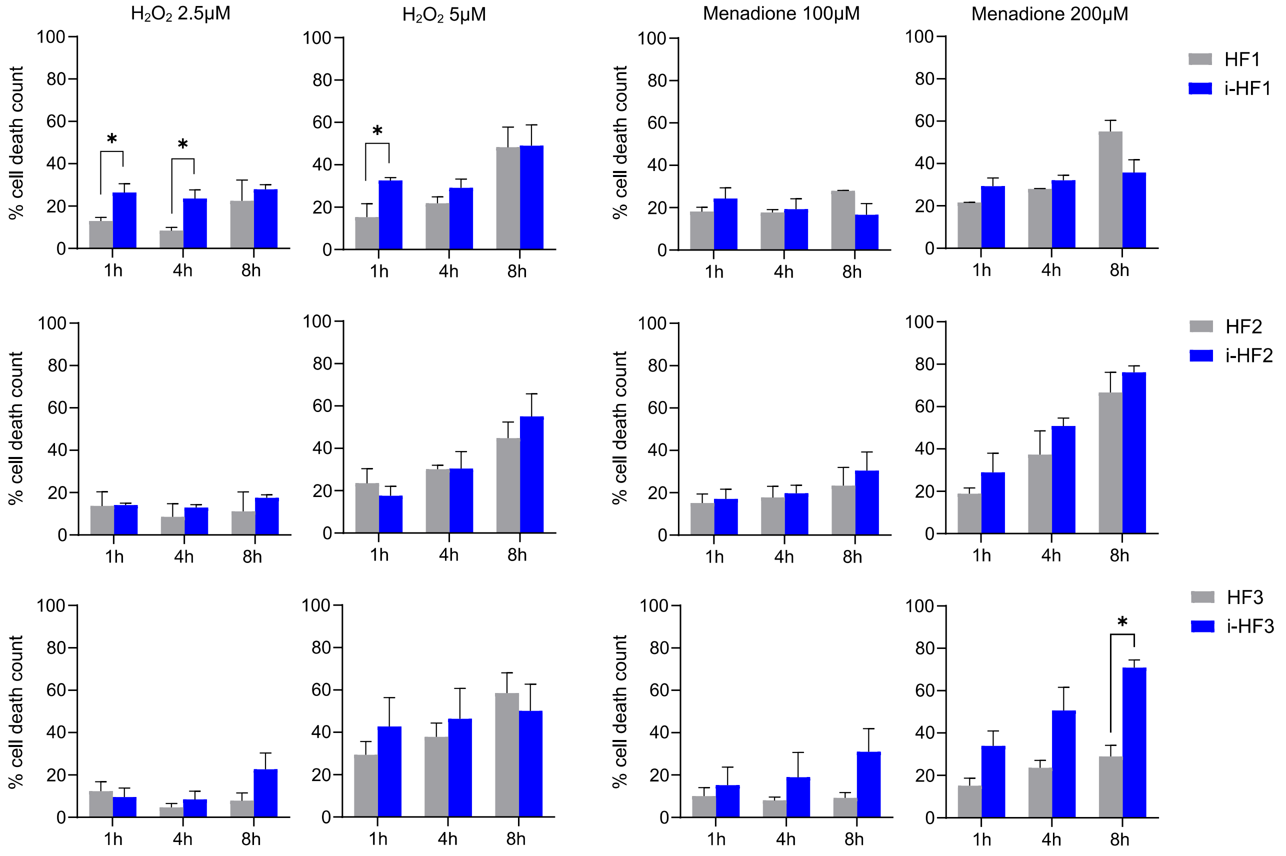

Figure S4. i-HF from donors 1 and 3 exhibit increased sensitivity to oxidative stress. Percentage of cell death caused by two different doses of H2O2 or menadione, two known inducers of oxidative stress. Cell death was assessed by propidium iodide staining using flow cytometry on the indicated HF and i-HF lines at different timepoints (1h, 4h and 8h) after cells were treated for 1h with the indicated reagent. Multiple unpaired t tests were applied for each timepoint. Shown is mean±SEM of n=3 independent experiments.

Supplementary Table S1: List of antibodies

Antibodies	Supplier reference	Dilution	Application
TRA1-60	Life technologies - A24881	1:500	IF
SSEA4	Life technologies - A24881	1:500	IF
Vimentin	Invitrogen – MA5-16409	1:200	IF
FSP-1	Sigma – F4771	1:200	IF
hTERT	Santa cruz – sc-393013	1:200	IF
Iso PE Rat IgG2bk (RTK4530)	Biolegend - 400608		FACS
Iso FITC IgG2ak (MOPC-173)	Biolegend - 400208		FACS
Iso APC IgG1k (MOPC-21)	Biolegend - 400122		FACS
CD44 (IM7)	Biolegend - 103008		FACS
HLA-1 - FITC	Biolegend – cl W6/32 311404		FACS
CD73 - APC	Biolegend – cl AD2 344006		FACS
BrdU	BD – cl B44 347580	1:250	IF
P21	R&D – AF1047	1:1000	WB
p53	Santa Cruz - sc-126	1:1000	WB
Phosphor-p53 (Ser15)	Cell signaling – 9284S	1:1000	WB
CDK4	Cell signaling – cl D9G3E 12790S	1:1000	WB
p16	BD – 51-1325GR	1:500	WB
Vinculin	Santa Cruz – sc-73614	1:5000	WB
yH2Ax (Ser139)	EMD Millipore – cl JBW301	1:250	IF
53BP1	Novus – NB100-304	1:1500	IF

Supplementary Table S2: List of primers

18S Fw	TCAACTTTCGATGGTAGTCGCCGT
18S Rev	TCCTTGGATGTGGTAGCCGTTTCT
CDKN1A Fw	GGCAGACCAGCATGACAGATT
CDKN1A Rev	GCGGATTAGGGCTTCCTCTT
GADD45A Fw	CTGGAGGAAGTGCTCAGCAAA
GADD45A Rev	GCACAACACCACGTTATCGG
Sce1 NHEJ Fw	AGGTCCCTGAGGCTACCTCCAGTTC
Sce1 NHEJ Rv	CATAGTGAGGAGTACCAGGACAG

Supplementary Table S3: NHEJ GFP construct sequence comprised between primers used for amplification.

tccctgaggctacctccagttctaaggctgcactccattctcacagccaggtctgctcagggcacagggcacagggtgaggcccttcact
ttcccggcatttaaacatagccctggaaagagaagctgacagttgcaaagctgctttgcttgccttggcttcagtggcccttctgacggtag
agtgacccacacaccacaatgaaggggaaagaggaccttctaatgcattcttagatatttcaacatgggtgtttccaagaagctttaggga
taaXcagggtaatctgcacgtctagggcgcagtagtccagggtttccttgatgatgtcatacttatcctgtcccttttttttttccacagctcgcg
gttgaggacaaactcttcgcggtctttccagtactcttggatcggaaacccgtcggcctccgaacggtaagagcctagcatgtagaactgg
ttcgacctgcagattaccctgttatXccctaaagcttgtcacagacccctcctgctctggtttctaagcatccccagaaatgtaacttgaaa
gtgttctgggagtgaccatggccttgtttccctctctggaactgtttgtaaactctgaccactgctgtcctggtactcctcactatg

aaa: Primers
aaa: Intron sequence
aaa: I-Sce1 recognition sequence
aaa: Adenoviral exon sequence

X: Expected I-Sce1 cut sites
